# Good response to plasmapheresis and mycophenolate mofetil in a patient with anti-synthetase syndrome associated with peripheral neuritis and severe prosthetic aortic valve stenosis

**DOI:** 10.1186/s43166-022-00127-z

**Published:** 2022-05-05

**Authors:** Zahraa Nour Eldine Ismail, Elham Azmy Abdulkader Abdellatif

**Affiliations:** Department of Physical Medicine, Rheumatology and Rehabilitation, Faculty of Medicine, Suez Canal University, Ismailia, Egypt

**Keywords:** Idiopathic inflammatory myopathies, Peripheral neuropathy, Plasmapheresis, Anti-synthetase syndrome

## Abstract

**Background:**

Idiopathic inflammatory myopathies (IIM) are a disease complex that encompasses several distinctly acquired muscle illnesses. Anti-synthetase syndrome is a subset of IIM that is characterized by the presence of antibodies against aminoacyl-tRNA synthetases (ARS). This syndrome has a characteristic phenotype of IIM. Anti-synthetase syndrome was rarely associated with peripheral nerve involvement and endocarditis.

**Case presentation:**

We report a 46-year-old female patient with a history of mitral and aortic valve replacement for seven years and on warfarin, presented with symmetrical muscular weakness, generalized edema, progressive dyspnea, dysphagia, fever, fatigue, myalgia, and polyarthralgia. The motor power grading was 2 in proximal muscles of upper limbs and 3 in the distal muscles involving hands and wrists, and it was 0 in proximal muscles in lower limbs and 2 in distal muscles involving ankle and toes movements. Also, her oxygen saturation decreased remarkably. Echocardiography revealed that the patient had severe stenosis (70%) of the prosthetic aortic valve. Electrophysiological studies showed axonal polyneuropathy with average F wave latencies. She was diagnosed with anti-synthetase syndrome for elevated muscle enzymes, interstitial lung disease (ILD), mechanic’s hands, fever, polyarthralgia, and positive anti-Jo-1 antibody. There was a significant improvement with plasmapheresis, mycophenolate mofetil (MMF), and high-dose prednisolone.

**Conclusions:**

To the best of our knowledge, this is a case of anti-synthetase syndrome accompanied by neuropathic involvement and cardiac valve prosthetic stenosis. These were reported as an unusual presentation of the anti-synthetase syndrome. The significant improvement with plasmapheresis gives us a treatment choice for similar critical cases.

## Background

Autoantibodies are a feature of several systemic autoimmune rheumatic disorders, including idiopathic inflammatory myopathies (IIM). iIIM autoantibodies are classified as myositis specific (MSA) or myositis associated (MAA) based on their specificity [1]. Autoantibodies against aminoacyl-tRNA synthetases (ARS) are the most prevalent MSA and can be seen in 25–35% of individuals [2]. The most frequent is the anti-Jo-1 antibody (anti-histidyl-tRNA synthetase antibody), which is identified in 15–30% of individuals with polymyositis (PM) and 60–70% of those with interstitial lung disease (ILD) [3]. The presence of ARS and other autoantibodies has become an important criterion for IIM classification and diagnosis, and it is increasingly being utilized to establish clinically distinct IIM subgroups [4].

Anti-synthetase syndrome is a systemic autoimmune illness that includes inflammatory myopathy, arthritis or arthralgias, interstitial lung disease (ILD), fever, Raynaud’s phenomenon, and mechanic’s hands. It is significantly linked to the development of anti-ARS antibodies, which are implicated in the etiology of muscle and pulmonary damage and directly correspond with illness severity [5]. Anti-synthetase syndrome has many clinical characteristics with PM, but histologically, the inflammatory component is similar to dermatomyositis (DM) [6]. Peripheral nervous system involvement and valvular heart disease in anti-synthetase syndrome are uncommon and poorly understood.

## Case presentation

A 46-year-old female patient has had a history of mitral and aortic valve replacement for seven years and is on warfarin. She was presented 6 months ago with intermittent low-grade fever, generalized weakness, and dyspnea. Weakness was gradual, progressive, more proximal than distal, and more in her lower limbs than the upper. She had great difficulty getting up from bed or a chair and climbing stairs until she was bedridden in 1 month. Dyspnea was exertional, with a progressive course, and accompanied by any activity. The patient also had myalgia and polyarthralgia involving all joints proximally and distally. There were no sensory or sphincteric abnormalities, arthritis, Raynaud’s phenomenon, skin lesions, or proceeding infections.

**Fig. 1:**
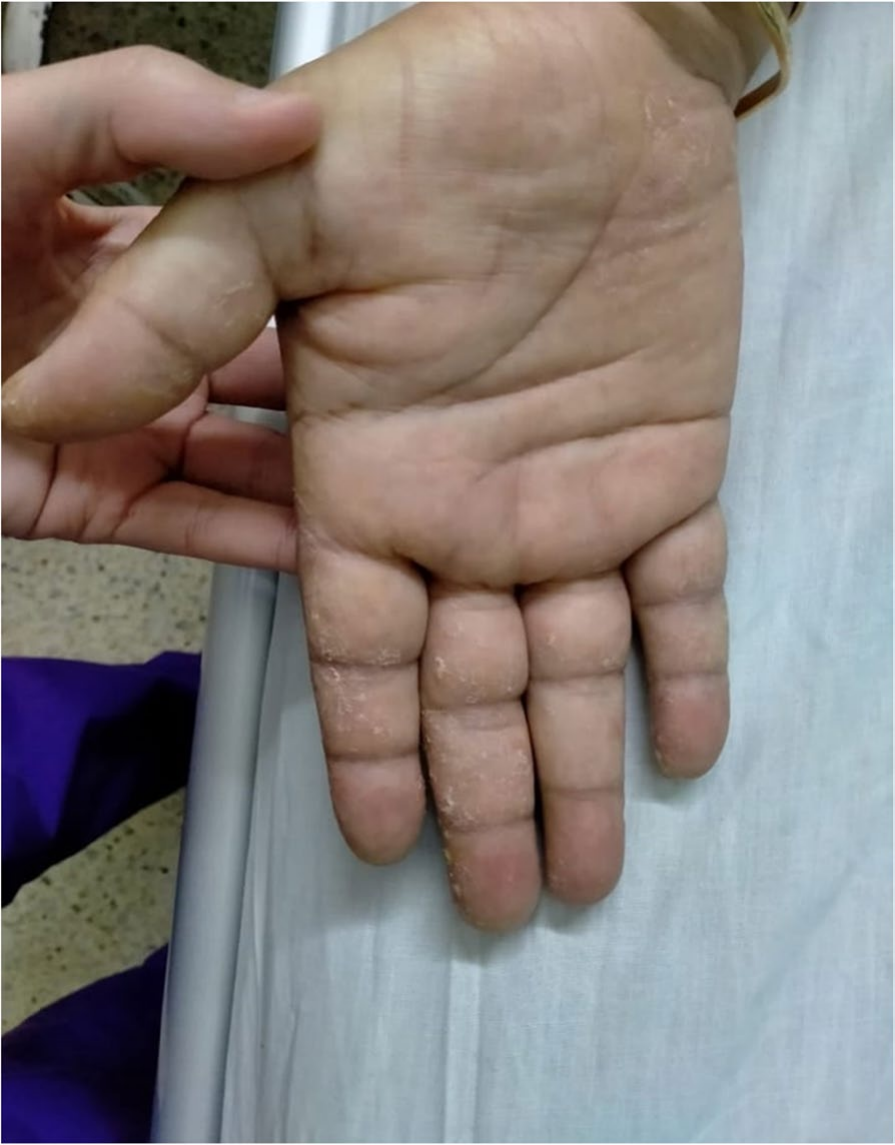
Mechanic’s hand

One month later, she developed dysphagia that was initially to solids, and then exacerbated till it also involved liquids causing feeding difficulty. Also, the dyspnea progressed to be at rest; the weakness was extreme and disabling. The patient was admitted for diagnosis of her condition. On our examination at admission, she was conscious, alert, dyspneic, and had generalized edema. The respiratory rate was elevated (23 breaths per minute), blood pressure was normal, and pulse was faint (60 beats per minute). There were hypotonia and hyporeflexia. No fasciculations, pathological reflexes, signs of lateralization, or increased intracranial tension. The sensations were normal. Arterial blood gases, pH, and oxygen saturation were within the normal range. There were bilateral basal inspiratory crackles and mechanic’s hands, as shown in Fig. 1. The motor power grading was 2 in proximal muscles of upper limbs, 3 in the distal muscles in hand and wrist; however, it was 0 in proximal muscles in lower limbs, and 2 in distal muscles of ankle and toes dorsiflexors and plantar flexors.

After a few days of admission, the dyspnea was severe until oxygen saturation was extremely low (between 70 and 80 on room air, then the patient had oxygen therapy without elevation of the saturation). The generalized edema was increased significantly. The patient was admitted to the coronary care unit (CCU) for monitoring and oxygen therapy.

Regarding the patient’s investigations, there were extremely elevated muscle enzymes as creatine phosphokinase (CPK), creatine kinase-myoglobin binding (CK-MB), alanine aminotransferase (ALT), and aspartate aminotransferase (AST). Also, serum aldolase, lactic dehydrogenase (LDH), and ferritin were elevated, as shown in Table 1. Antinuclear autoantibodies (ANA) (1/80 titer and speckled in pattern), as well as the anti-Jo-1 antibody, were positive. There were also leukocytosis and thrombocytosis. Dyslipidemia was accidentally discovered and she started 40 mg atorvastatin and 10 mg ezetimibe daily.

**Table 1:** Investigations before and after treatment

Investigations (normal range)	Before treatment	After 5 months of treatment
**ESR in the first hour**	Over 100 mm	22 mm
**CRP (0−5)**	36 mg/L	4.1 mg/L
**CPK (33−211)**	12970 U/L	254 U/L
**CK-MB (0−5)**	179 ng/ml	30.3 ng/ml
**Troponin (0−0.04)**	5 μg/L	0.04 μg/L
**ALT (0−33)**	490 U/L	49 U/L
**AST (0−32)**	373 U/L	37 U/L
**Aldolase (1−7.5)**	30.5 U/L	9.5 U/L
**LDH (140−280)**	1231 U/L	190 U/L
**Ferritin (24−300)**	2376 ng/mL	150 ng/mL
**Albumin (3.5−5.5)**	2.6 g/dL	3.9 g/dL
**HB (11.5−15.5)**	10.9 g/dl	12.4 g/dl
**TLC (4−11)**	19.02 thousand/cmm	7.2 thousand/cmm
**Platelets (150−450)**	582 thousand/cmm	264 thousand/cmm
**Total cholesterol (up to 200)**	207 mg/dL	153 mg/dL
**Serum triglycerides (0−150)**	173 mg/dL	150 mg/dL
**HDL cholesterol (40−60)**	37 mg/dL	29 mg/dL
**LDL cholesterol (0−100)**	135 mg/dL	69 mg/dL

*ESR* Erythrocyte sedimentation rate, *CPK* Creatine phosphokinase, *CK-MB* Creatine kinase-myoglobin binding, *ALT* Alanine aminotransferase, *AST* Aspartate aminotransferase, *LDH* Lactic dehydrogenase, *HB* Hemoglobin, *TLC* Total leucocyte count, *CRP* C-reactive protein, *HDL* High-density lipoprotein, *LDL* Low-density lipoprotein, *mg/L* Milligrams per liter, *U/L* Units per liter, *ng/ml* Nanograms per milliliter, *μg/L* Microgram/liter, *g/dL* Grams per deciliter, *mg/dL* Milligrams per deciliter

The thyroid hormones, 24-h urine protein, creatinine, complement component 3 (C3), complement component 4 (C4), anti-double-stranded DNA (anti-ds DNA), antiphospholipid antibodies, rheumatoid factor (RF), anti-cyclic citrullinated peptides (anti-CCP), ribonucleoprotein antibody (anti-RNP), anti-smith antibody, anti-smooth muscle antibody, and tumor markers were all within normal range. The polymerase chain reaction (PCR) test for COVID-19 was negative.

Echocardiography revealed severe stenosis (70%) of the prosthetic aortic valve. However, the prosthetic mitral valve was utterly patent. The computerized tomography (CT) on the chest showed a ground-glass appearance in basal lung lobes with increased peribronchovascular markings, interlobular septal thickening, pulmonary congestion, and cardiomegaly.

**Fig. 2:**
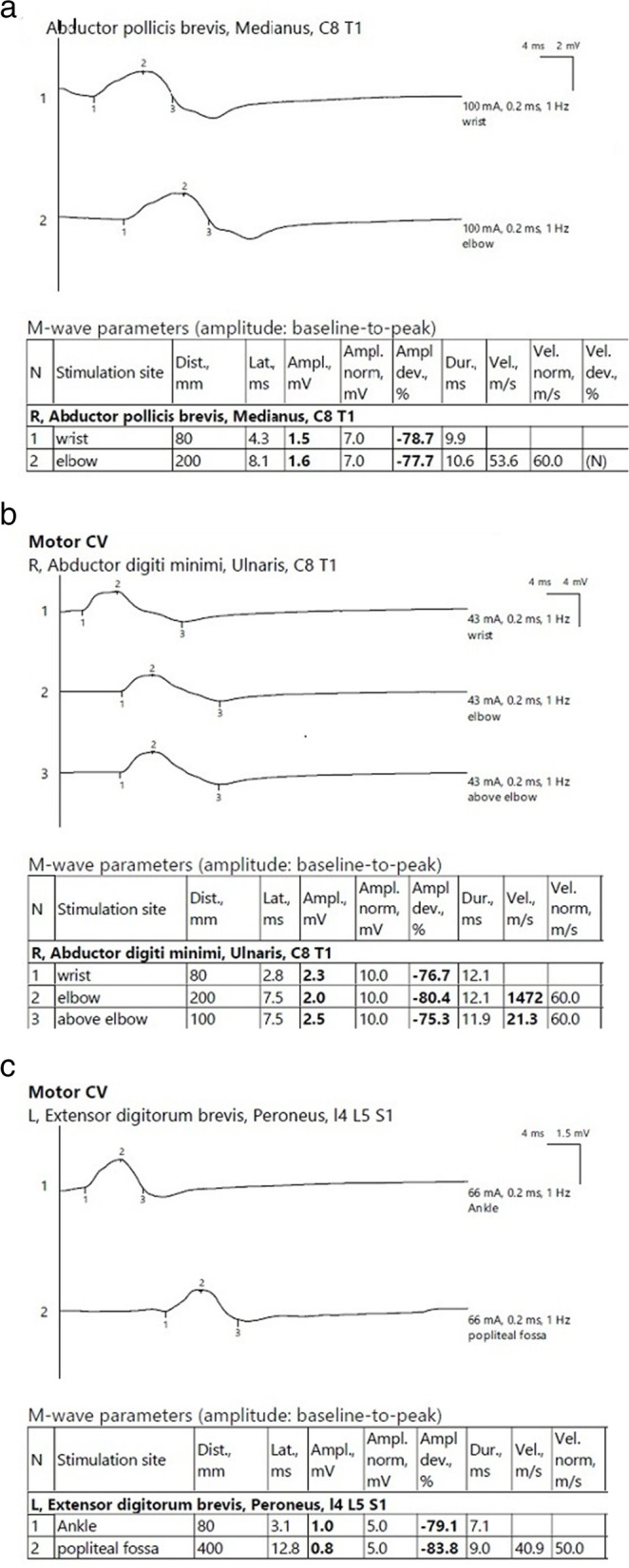
**a** Axonal neuropathy of right median nerve at admission before starting treatment. **b** Axonal neuropathy of right ulnar nerve at admission before starting treatment. **c** Axonal neuropathy of left common peroneal nerve before treatment

**Fig. 3:**
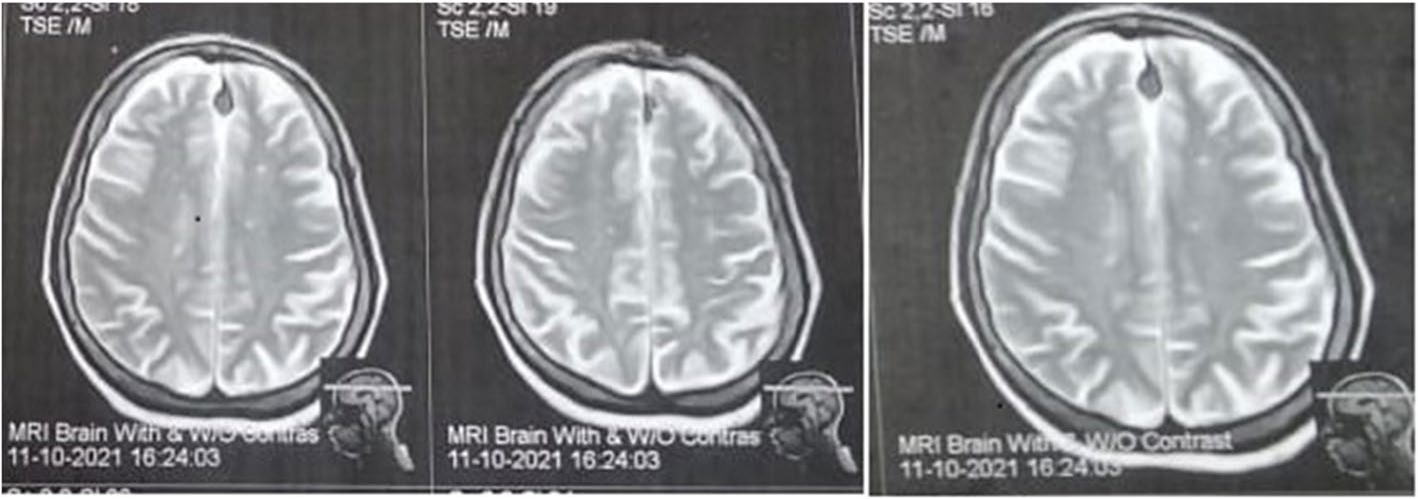
Multiple parietal deep white matter foci suggestive of vasculitis versus microangiopathy

The nerve conduction study showed axonal polyneuropathy, as shown in Fig. 2a–c, and surface electrode electromyography showed large amplitudes and duration motor units. We could not do a muscle biopsy as the patient was on warfarin. Magnetic resonance imaging (MRI) brain with contrast revealed abnormally high signal intensity white matter lesions in the high parietal regions, as shown in Fig. 3. These lesions were suggestive of small vessel vasculitis versus microangiopathy.

She was diagnosed with the anti-synthetase syndrome. We decided to start prednisolone only instead of pulse methylprednisolone for fear of sepsis as she had fever and leukocytosis; also, her general condition was unstable. She had 60 mg prednisolone that was tapered to 40 mg in 5 days. She also had three doses of intravenous immunoglobulins (IVIG) of 24,000 mg daily with mild improvement in the general condition and oxygen saturation after the first session, then no more progress.

We decided to elevate the prednisolone dose again to 60 mg daily and added mycophenolate mofetil (MMF) 3 gm daily. The condition started to improve regarding dyspnea, oxygen saturation (average 90 with nasal cannula 5 L per minute), generalized edema, and dysphagia after about 7 days of the beginning of MMF with high-dose prednisolone. The motor power improved minimally. We decided to do plasmapheresis for the severity of the condition and to accelerate recovery. She had day after day plasmapheresis sessions (four sessions) with significant improvement of her oxygen saturation (became 95 in room air) and muscle power grading (became 4+ in distal muscles (both hands and elbows) and 3 in proximal muscles, it was 3 in both lower limbs proximal muscles while 4 + in distal muscles as plantar flexors, dorsiflexors, evertors, and invertors). The CPK also decreased to 890 U/L.

**Fig. 4:**
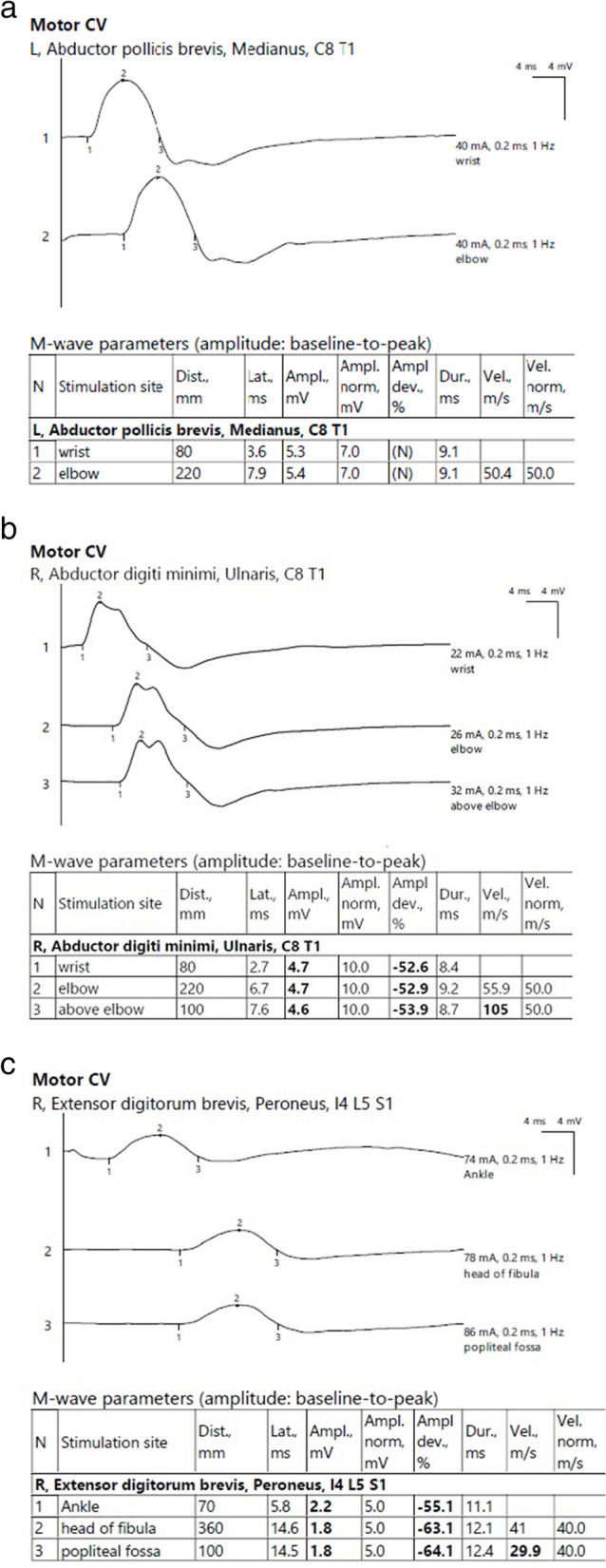
**a** Normal electrophysiological study of left median nerve after 4 months of treatment. **b** Normal electrophysiological study of right ulnar after 4 months of treatment. **c** Normal electrophysiological study of right common peroneal after 4 months of treatment

She was discharged on 60 mg prednisolone and 3 gm mycophenolate mofetil. After 2 months, we started steroid tapering when the condition improved clinically, and serum CPK was 500. We created an alternate-day regimen of steroid tapering. Now she is on 30 mg prednisolone daily, alternating with 20 mg. Now the dyspnea and weakness have improved, CPK is 254, muscle power grading is 5 in both proximal and distal muscles; nerve conduction studies of all nerves are average, as shown in Fig. 4a, b; the prosthetic aortic valve is ultimately patent, and CT chest is completely normal. Table 1 shows the investigations before and after 5 months of treatment. She just experiences myalgia in her proximal muscles after effort.

## Discussion

This is a case of anti-synthetase syndrome. This diagnosis was made based on the patient's presentation with myositis, fever, arthralgia, mechanic’s hands, and ILD, in addition to the presence of anti-Jo1 as one of the antibodies against ARS. These are all the features of the anti-synthetase syndrome. There was a previous diagnosis of Guillain-Barre syndrome as she had polyneuropathy, and the weakness was more in the lower limbs than the upper. There was no proceeding infection; the F waves of all nerves were of average latencies, so this diagnosis cannot be considered.

The present polyneuropathy is a rare presentation of anti-synthetase syndrome. It was reported once in PM [7] and also reported in a few DM cases [8, 9]. The pathophysiology of polyneuropathy can be related to severe disease presentation. Also, the progressing generalized edema can cause peripheral neuropathy through compression of peripheral nerves. We can connect the accompanied peripheral neuropathy to a small vascular disease or vasculitis involving the vasa nervorum, causing peripheral nerves ischemia and peripheral neuropathy, as there was suspicious small vessel vasculitis in the brain MRI. Overall, severe cases with IIM can present with peripheral neuropathy, and polyneuropathy cannot exclude the diagnosis of polymyositis.

Regarding the valvular affection in the case, our case had a severe prosthetic aortic valve that may have a role in pulmonary congestion, cardiomegaly, and dyspnea. Valvular heart affection is not previously reported in anti-synthetase syndrome or IIM. The improvement after treatment increases the probability of its relation to the disease. Its proposed mechanism can be endocarditis, which can happen besides myocarditis in those patients. Also, the disease can accelerate pannus formation and cause prosthetic valve stenosis.

Regarding the management of the patient, we did not start pulse methylprednisolone and intravenous cyclophosphamide as the patient was suspected of having infections for the presence of leukocytosis. So, we decided to give oral high-dose steroids (prednisolone) and MMF. MMF was reported as a successful initial treatment [10] and effective in severe refractory anti-synthetase syndrome [11].

We decided plasmapheresis as it was reported before in the treatment of refractory patients with PM and DM that did not respond well to immunosuppressants [12, 13]. Plasmapheresis can remove circulating autoantibodies, immune complexes, complement components, cytokines, and adhesion molecules and sensitize antibody-producing cells to immunosuppressive drugs to be beneficial in severe autoimmune disease [14]. This good response gives us fast and effective treatments for similar disease presentations.

## Conclusion

The systemic autoimmune disease must be considered before diagnosing peripheral neuropathy as it can occur in most autoimmune diseases. Anti-synthetase syndrome patients may have peripheral nervous system involvement and cardiac valves involvement. A combination of plasmapheresis and MMF effectively manages anti-synthetase syndrome with peripheral polyneuropathy and valvular heart affection.

## Data Availability

All clinical data used for writing this case report are included in this article (figures).

## Abbreviations

IIM: Idiopathic inflammatory myopathies
ILD: Interstitial lung disease
PM: Polymyositis
DM: Dermatomyositis
ARS: Aminoacyl-tRNA synthetases
MSA: Myositis specific antibodies
MAA: Myositis associated antibodies
C3: Complement component 3
C4: Complement component 4
Anti-ds DNA: Anti-double-stranded DNA
RF: Rheumatoid factor
Anti-CCP: Anti-cyclic citrullinated peptides
Anti-RNP: Ribonucleoprotein antibody
CT: Computerized tomography
COVID-19: Coronavirus infection of 2019
PCR: Polymerase chain reaction
CPK: Creatine phosphokinase
CK-MB: Creatine kinase-myoglobin
ALT: Alanine aminotransferase
AST: Aspartate aminotransferase
LDH: Lactic dehydrogenase
ANA: Antinuclear autoantibodies
HB: Hemoglobin
TLC: Total leucocyte count
CCU: Coronary care unit
MMF: Mycophenolate mofetil
IVIG: Intravenous immunoglobulin
